# Time-Resolved Secondary Metabolite Profiling of Seeded and Seedless Ougan at Commercial Harvest Maturity

**DOI:** 10.3390/cimb48060596

**Published:** 2026-06-05

**Authors:** Quan Zhao, Peian Zhang, Yang Song, Fayong Li, Yingyao Liu, Jun Chen, Dongfeng Liu

**Affiliations:** Zhejiang Institute of Subtropical Crops, Wenzhou 325005, China; zhaoq@zaas.ac.cn (Q.Z.); 2018204002@njau.edu.cn (P.Z.); songy@zaas.ac.cn (Y.S.); lify@zaas.ac.cn (F.L.); yingyao@u.nus.edu (Y.L.); 15968220763@163.com (J.C.)

**Keywords:** Ougan, fruit quality, secondary metabolites, flavonoids, metabolomics, UPLC-MS/MS

## Abstract

Ougan (*Citrus suavissima* Hort. et Tanaka) is valued for its distinctive sweet–bitter flavor and nutritional properties; however, tissue-resolved metabolic differences between two cultivar forms (seeded and seedless) of Ougan (*C. suavissima*) remains poorly understood. In this study, a comprehensive UPLC-MS/MS-based metabolomic analysis was conducted on peel (SP and NP), pulp (SF and NF), segment membrane (SM and NM) and seed tissues (SS, from seeded fruit only) of seeded and seedless Ougan fruits. A total of 1333 metabolites were annotated, with flavonoids (48.53%) and phenolics (12.25%) representing the predominant compound classes. Tissues specificity was the primary determinant of metabolic variation, with peel and segment membrane tissue showing relatively high abundance (fold change ≥ 2, |Log_2_FC| ≥ 1) of phenylpropanoid- and flavonoid-derived metabolites. Comparative analysis between seeded and seedless tissues revealed significant modulation of phenylpropanoid biosynthesis, flavonoid biosynthesis, phenylalanine metabolism, and related secondary metabolite pathways. Seeded tissues showed a higher relative abundance of selected flavonol glycosides (6-hydroxykaempferol-3,6-O-diglucoside), hydroxycinnamic acid derivatives, and santhocyanin-related compounds, whereas seedless tissues showed higher relative abundance of selected flavanones and malonylated flavonoid glycosides. Seeds were characterized by high limonin content, consistent with limonoid-associated bitterness chemistry. Overall, our findings provide a tissue-resolved metabolomic framework for understanding quality-associated secondary metabolite variation in mature Ougan fruit.

## 1. Introduction

Citrus fruits represent a major component of human diets worldwide and are widely investigated due to their rich composition of bioactive metabolites and associated health-promoting properties [1]. Species within the genus Citrus, including *Citrus limon*, *Citrus reticulata*, and *Citrus latifolia*, are well documented as important sources of phytochemicals such as ascorbic acid, limonoids, flavonoids, phenolic acids, and alkaloids [2]. These metabolites contribute not only to nutritional value but also to fruit sensory characteristics, including color, aroma, and taste [3,4]. Ougan (*Citrus suavissima* Hort. et Tanaka) is a traditional loose-skin mandarin-type citrus endemic to Wenzhou, Zhejiang Province, China, where it has been cultivated for over 400 years. Classified within the family Rutaceae, it exists in two principal cultivar forms: the original seeded Ougan (10–20 seeds per fruit) and a naturally occurring seedless mutant derived through bud-sport selection [5]. Both share the characteristic sweet–bitter flavor attributed to flavanone glycosides and limonoids, but the seedless variety commands higher fresh-market value due to consumer preference for convenience. Ougan breeding has shifted toward seedless varieties, with current priorities including improved soluble solids content, reduced bitterness, and enhanced shelf life. Functional breeding targeting flavonoid- and limonoid-rich cultivars is an emerging direction given their established bioactivities [4,6]. Understanding tissue-specific metabolite differences between seeded and seedless phenotypes is therefore directly relevant to quality-oriented Ougan breeding. To our knowledge, no tissue-resolved metabolomic comparison of the two forms has been reported.

Among citrus secondary metabolites, flavonoids are the predominant phenolic class and are key determinants of fruit pigmentation and flavor [3]. Citrus flavonoids, including flavanones, flavones, flavonols, and polymethoxyflavones, exhibit strong antioxidant activity and have been associated with anti-inflammatory, anticancer, and cardioprotective effects [7]. Their bioactivity is largely attributed to free radical scavenging capacity, metal ion chelation, and modulation of enzymatic pathways [8]. These triterpenoid compounds, which belong to the flavonoid, occur as both water-soluble glucosides and bitter aglycones, with limonin and nomilin being among the most abundant representatives [8]. Limonoids significantly influence bitterness perception and have been reported to possess antimicrobial, immunomodulatory, antiaging, and anticancer properties [9].

Advances in analytical technologies have improved the characterization of citrus metabolomes. Traditional liquid chromatography coupled with diode array detection (LC-DAD) has been widely used for targeted flavonoid and limonoid quantification [10]; however, its reliance on authentic standards limits comprehensive profiling. More recently, liquid chromatography coupled with high-resolution tandem mass spectrometry (LC-MS/MS or LC-MS/HRMS) has emerged as a powerful tool for untargeted and semi-targeted metabolomics, enabling accurate mass determination and MS/MS spectral matching against extensive databases [11]. This approach has been successfully applied to characterize metabolite profiles in various citrus species, including oranges, lemons, mandarins, limes, and grapefruit [11,12,13].

Ougan (*Citrus suavissima* Hort. et Tanaka), a locally important citrus cultivar, is valued for its distinctive sweet and bitter flavor and nutritional quality [14]. However, while tissue-specific metabolite distributions have been characterized in related citrus species [11,12,13], limited information is available regarding how fruit phenotype differences, particularly between seeded and seedless cultivar forms, influence secondary metabolite composition across tissues in *Citrus suavissima*. Therefore, the objective of the present study was to perform comprehensive UPLC-MS/MS-based metabolomic profiling of peel, pulp, segment membrane, and seed tissues of seeded and seedless Ougan fruits at maturity. Specifically, we aimed to (i) characterize the tissue-resolved secondary metabolite landscape, (ii) identify tissue-associated differentially accumulated metabolites between seeded and seedless fruits, and (iii) determine enriched pathways, particularly those related to phenylpropanoid and flavonoid biosynthesis, associated with quality-associated chemical variation in mature Ougan fruit.

## 2. Materials and Methods

### 2.1. Experimental Site and Sampling

According to local picking habits and Ougan ripening conditions, the fruits were collected on 25 November 2024 from the Gaotianming Ougan plantation in Wenzhou city (120.70° E, 27.95° N), China. The experimental place’s climate is classified as a subtropical monsoon climate, with an annual average temperature of 19.4 °C and average annual precipitation of 1793 mm. The soil type of the experimental field was plain paddy soil. The soil properties of the experimental fields, i.e., pH, organic matter, available nitrogen, available phosphorus, available potassium, exchangeable calcium and exchangeable magnesium, were 5.22, 15.0 g kg^−1^, 94.3 mg kg^−1^, 134.2 mg kg^−1^, 90.9 mg kg^−1^, 815.8 mg kg^−1^ and 325.6 mg kg^−1^, respectively. All trees in the orchard received uniform background fertilization with 1.5 kg of ternary NPK compound fertilizer and 10 kg of organic fertilizer per tree per year, following the standard management practices of the base. Other agronomic practices, including irrigation, pruning, and pest management, were conducted uniformly.

**Figure 1:**
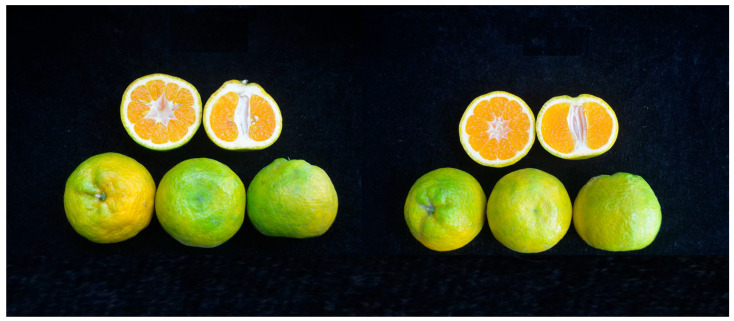
Seeded Ougan (**left** side) and seedless Ougan (**right** side) at maturity. Seeded Ougan with an average weight of 132 g, an average longitudinal diameter of 58.8 mm, and an average transverse diameter of 68.7 mm. Seedless Ougan with an average weight of 117 g, an average longitudinal diameter of 56.4 mm, and an average transverse diameter of 64.8 mm.

In the orchard, six trees aged 15 years were randomly selected for each variety. Seeded Ougan and seedless Ougan are different varieties, with the latter being a seedless mutant variety of the former. Mature fruits were collected 225 days after flowering when the fruit peel exhibited a yellow–green color (Figure 1).

Fruit collection was performed during a single harvest season (November 2024), representing the first year of this project. Since Ougan is harvested once per year and fruit development and ripening are tightly coordinated within the orchard, a single-season design was adopted to establish a tissue-resolved metabolomic baseline at commercial harvest maturity.

Random sampling was conducted on the mature fruits from the east, south, west, and north directions of these six trees. Thirty representative fruits that were disease-free and of normal size were collected from each variety. The collected seeded Ougan fruits were divided into four different types of samples: peel (including the oil gland layer and white skin layer), segment membrane, pulp and seeds (SP, SM, SF, and SS, respectively); the collected seedless Ougan fruits were divided into three different types of samples, namely the peel, segment membrane, and pulp (SP, SM, and SF). For each tissue type, three independent biological replicates were analyzed. Each replicate consisted of pooled material derived from multiple fruits collected from representative trees within the orchard to minimize intra-tree variability. All samples were then freeze-dried, crushed separately and stored at −80 °C for future use.

### 2.2. Reagents

Methanol (MeOH), acetonitrile (ACN), and formic acid were LC-MS grade and obtained from commercial suppliers certified for mass spectrometry applications. Ultrapure water was generated using a Milli-Q purification system. All other analytical reagents were of chromatographic grade. Internal standards supplied within the Metware targeted metabolomics platform were used for quality control and signal normalization [11].

### 2.3. Instrument and Software

Metabolomic profiling was conducted using an ExionLCTM AD ultra-performance liquid chromatography (UPLC) system coupled to a triple quadrupole linear ion trap mass spectrometer equipped with an electrospray ionization (ESI) source (AB Sciex). Data acquisition was performed in multiple reaction monitoring (MRM) mode. Analyst 1.6.3 software was used for instrument control and data acquisition, and MultiQuant 3.0.2 software was used for peak integration and relative quantification [15]. Multivariate statistical analysis was performed in R (v 4.5.2) using the prcomp function in PCA and the ComplexHeatmap package (v 2.26.1) for clustering visualization.

### 2.4. Metabolite Extraction

Fruit tissues (peel, pulp, segment membrane, and seeds) were freeze-dried and ground into fine powder using a mixer mill (30 Hz, 1.5 min). Approximately 50 mg of powdered sample was extracted with 1200 μL of pre-chilled 70% methanol (4 °C) containing internal standards. Internal standards provided within the Metware platform consisted of a mixture of isotopically labeled compounds (L-2-chlorophenylalanine and other stable isotope-labeled analogs) used for signal normalization and instrument performance monitoring. Samples were vortexed every 30 min for six cycles and then stored at 4 °C overnight to ensure complete extraction. After centrifugation at 12,000 rpm for 3 min, the supernatant was filtered through a 0.22 μm membrane filter before UPLC-MS/MS analysis. Quality control (QC) samples were prepared by pooling equal aliquots from all extracts and injected periodically to monitor instrument stability and reproducibility [16].

### 2.5. UPLC-MS/MS Analysis

Chromatographic separation was achieved using a C18 reversed-phase column (ACQUITY UPLC HSS T3; Waters Corp., Milford, MA, USA) (1.8 μm particle size, 2.1 × 100 mm) maintained at 40 °C. The mobile phases consisted of water containing 0.1% formic acid (phase A) and acetonitrile containing 0.1% formic acid (phase B). The flow rate was 0.35 mL min^−1^, with a gradient program increasing phase B from 5% to 95% over 9 min, followed by column equilibration, resulting in a total run time of 14 min. Mass spectrometry was performed in positive and negative ion modes. The ion spray voltage was +5500 V in positive mode and −4500 V in negative mode, and the source temperature was 550 °C. Nitrogen was used as the nebulizer and auxiliary gas. Optimized declustering potential (DP) and collision energy (CE) parameters were applied for each metabolite, and detection and quantification were conducted using MRM transitions specific to each compound [17].

### 2.6. Metabolite Identification and Quantification

Metabolite annotation was performed by matching MS/MS spectra against the self-built MWDB (Metware Database), which integrates reference spectra and compound annotations. Redundant signals, including isotope peaks, adduct ions (e.g., Na^+^, K^+^, and NH_4_^+^), and in-source fragments, were removed during data processing. Relative quantification was performed based on peak area integration of MRM chromatograms, and peak areas were normalized across samples to reduce analytical variation [18].

### 2.7. Data Processing and Statistical Analysis

Raw peak area data were log_2_-transformed and unit variance-scaled prior to statistical analysis. Principal component analysis (PCA) was performed using the R function prcomp (scale = TRUE). Differentially accumulated metabolites (DAMs) between comparison groups were identified based on fold-change (FC) thresholds and statistical significance. KEGG pathway annotation and enrichment analysis were performed to identify significantly altered metabolic pathways. Throughout this manuscript, the term ‘higher relative abundance’ refers to a statistically meaningful difference defined by FC thresholds (FC ≥ 2 or ≤ 0.5; |Log_2_FC| ≥ 1) applied to log_2_-transformed MRM peak area values, which represent relative rather than absolute metabolite concentrations. As individual per-metabolite *p*-values are not generated by MRM-based targeted metabolomics platforms, statistical significance of overall group differences was confirmed by PCA separation and hierarchical cluster analysis.

## 3. Results

### 3.1. Comparative Metabolomic Profiling of Seeded and Seedless Ougan Fruits

**Figure 2:**
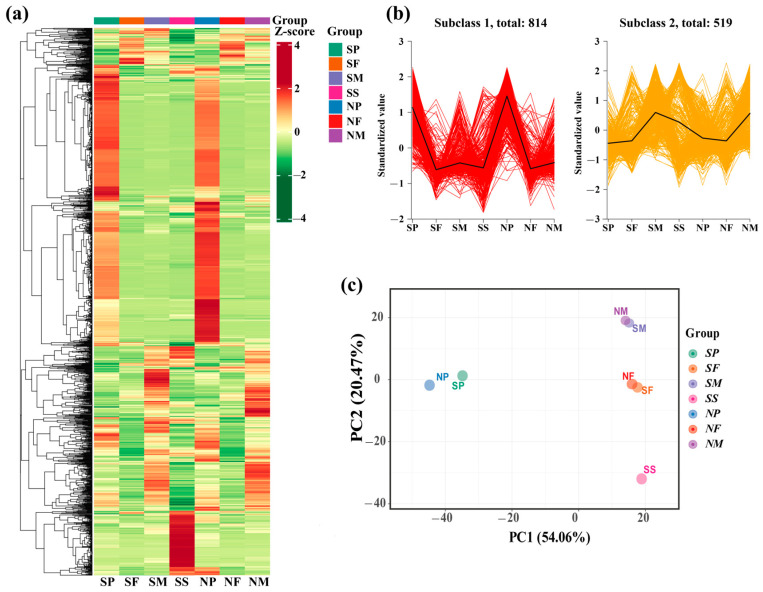
Metabolomic profiling of peel (SP and NP), pulp (SF and NF), segment membrane (SM and NM), and seeds (SS, from seeded fruit only) of seeded (S) and seedless (N) Ougan fruits at maturity. (**a**) HCA heatmap, (**b**) k-means clustering, and (**c**) PCA.

A comprehensive UPLC-MS/MS analysis was conducted on peel (SP and NP), pulp (SF and NF), segment membrane (SM and NM), and seed (SS) tissues of mature seeded and seedless Ougan fruits, resulting in the identification of 1333 metabolites (Table S1). HCA revealed clear tissue-dependent metabolic stratification (Figure 2a).

Peel samples (SP and NP) exhibited similar metabolic profiles that were clearly distinct from those of internal tissues. Segment membrane tissues (SM and NM) likewise displayed comparable metabolic patterns, as did pulp tissues (SF and NF). Seeds (SS) showed the most divergent metabolic profile. These results indicate that tissue specificity is the primary determinant of metabolic variation, whereas the seeded–seedless phenotype contributed additional tissue-matched differences. K-means clustering classified the detected metabolites into two major subclasses exhibiting distinct accumulation trends (Figure 2b). Subclass 1 (814 metabolites) displayed relatively higher accumulation in the peel of seedless fruit (NP), whereas seeds (SS) showed comparatively lower levels. In contrast, subclass 2 (519 metabolites) was predominantly enriched in segment membrane tissues (SM and NM), with lower accumulation in the peel of seeded fruit (SP). These patterns highlight strong spatial partitioning of secondary metabolites across Ougan fruit tissues. PCA revealed clear separation of samples according to tissue type and fruit phenotype (Figure 2c). The first two principal components explained 54.06% (PC1) and 20.47% (PC2) of the total variance, respectively, together accounting for 74.53% of the overall metabolic variation. PC1 primarily distinguished peel tissues (SP and NP; negative axis) from internal tissues and seeds (positive axis), whereas PC2 further separated membrane tissues (SM and NM) from pulp tissues (SF and NF).

**Figure 3:**
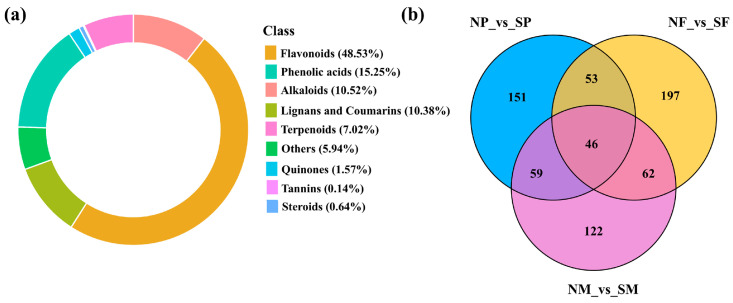
Metabolomic classification and differential metabolite distribution in peel (SP and NP), pulp (SF and NF), segment membrane (SM and NM), and seeds (SS) of seeded (S) and seedless (N) Ougan fruits at maturity. (**a**) Distribution of identified metabolite classes and (**b**) Venn diagram of comparison groups.

Metabolic classification identified nine major compound classes across Ougan tissues (Figure 3a). Flavonoids were the predominant group, accounting for 48.53% of total metabolites, followed by phenolic acids (15.25%), alkaloids (10.52%), and lignans and coumarins (10.38%). Steroids (0.64%) and tannins (0.14%) occurred at lower proportions. The predominance of flavonoids and phenolic acids supports their major contribution to Ougan fruit quality attributes and antioxidant potential. Venn diagram analysis of differentially accumulated metabolites (DAMs) among tissue-matched comparisons revealed both shared and tissue-specific metabolic differences (Figure 3b).

A total of 46 DAMs were common across the NP_vs_SP, NF_vs_SF, and NM_vs_SM comparisons, indicating a conserved metabolic signature associated with the seeded fruits. Among individual contrasts, NF_vs_SF exhibited the highest number of unique DAMs (197), followed by NP_vs_SP (151) and NM_vs_SM (122). Shared DAMs between NP_vs_SP and NM_vs_SM (59), and between NF_vs_SF and NM_Vs_SM (62), further indicate partial overlap of phenotype-associated metabolite differences across tissues. The top 20 DAMs (10 increased and 10 decreased) per pairwise comparison are summarized in Table S2 and ranked by absolute Log_2_ fold change. These findings demonstrate that tissue identity was the dominant determinant of the Ougan metabolome, while the seeded–seedless phenotype was associated with additional tissue-dependent shifts in secondary metabolite composition.

### 3.2. Differential Metabolite Analysis Between Seeded and Seedless Ougan Fruits

Across the 1333 annotated metabolites, pronounced tissue specialization was observed in mature Ougan fruit (Table S1). Peel tissues were enriched in protoalkaloids and other secondary metabolites. Seeded peel (SP) was dominated by synephrine (1.39 × 10^8^), followed by petrolactam (1.32 × 10^8^), and 5,7,5′-trimethoxy-3,4-methylenedioxyflavonoid (7.59 × 10^7^). In contrast, seedless peel (NP) showed the highest accumulation of petrolactam (1.23 × 10^8^), followed by synephrine (1.20 × 10^8^) and aracarpene 1 (1.03 × 10^8^). In pulp tissues, amide- and alkaloid-type metabolites were predominant. Seeded pulp (SF) showed the highest levels of amide-type N-(3-hydroxy-4-methoxyphenethyl)-4-hydroxybutanamide (7.46 × 10^7^), petrolactam (6.81 × 10^7^), and 4-nitrophenol (3.75 × 10^7^). Seedless pulp (NF) showed the highest abundance of petrolactam (7.39 × 10^7^), the same amide compound (4.94 × 10^7^), and hesperetin-7-O-glucoside (4.62 × 10^7^). Segment membrane tissues were enriched in flavonoids. Segment membrane (SM) had the highest levels of 4-nitrophenol (8.13 × 10^7^), synephrine (8.04 × 10^7^), and narirutin (6.90 × 10^7^), while seedless (NM) segment showed greater abundance of narirutin (8.36 × 10^7^), 4-nitrophenol (8.26 × 10^7^), and isoquercitrin (8.22 × 10^7^). Seeds (SS) were characterized by a high abundance of limonin (8.28 ×1 0^7^), sophorabioside (6.41 × 10^7^), and luteolin-7-O-(6-malonyl) glucoside (5.81 × 10^7^) (Table S1).

**Figure 4:**
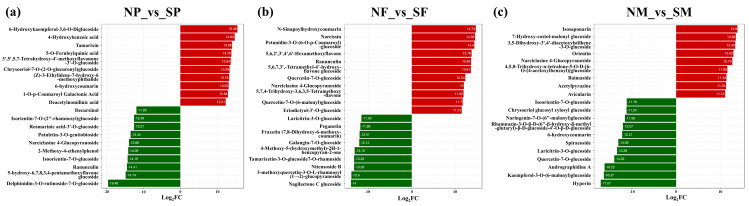
Bar plots showing the top differentially accumulated metabolites (log_2_ fold change) in the comparison groups: (**a**) NP_vs_SP, (**b**) NF_vs_SF, and (**c**) NM_vs_SM. Red bars indicate relatively higher abundance, and green bars indicate relatively lower abundance in seeded and seedless tissues.

Differential metabolite bar plots further illustrated phenotype-associated regulation in seeded tissues (SP, SF, and SM) compared with their seedless counterparts (Figure 4). In the NP_vs_SP comparison, 6-hydroxykaempferol-36-O-diglucoside showed the highest relative increase in seeded peel (SP; 15.36 log_2_FC), followed by 4-hydroxybenzoic acid (14.63 log_2_FC) and tamarixetin (13.98 log_2_FC), whereas delphinidin-3-O-rutinoside-7-O-glucoside exhibited the strongest relative decrease (−19.48 log_2_FC) (Figure 4a). In NF_vs_SF, N-sinapoylhydroxycoumarin was the most increased metabolite in seeded pulp (SF; 14.74 log_2_FC), followed by narcissin (14.56 log_2_FC), and petunidin-3-O-(6-O-p-coumaroyl)-glucoside (14.4 log_2_FC), while nagilactone C glucoside showed the greatest reduction (−14.00 log_2_FC) (Figure 4b). Similarly, in NM_vs_SM, isosaponarin displayed the highest relative increase in seeded membrane (SM; 13.90 log_2_FC), followed by 7-Hydroxy-costol-malonyl glucoside (13.56 log_2_FC), whereas hyperin exhibited the strongest relative decrease (−17.07 log_2_FC) (Figure 4c).

### 3.3. Tissue- and Genotype-Associated Metabolic Pathway Enrichment in Ougan Fruits

**Figure 5:**
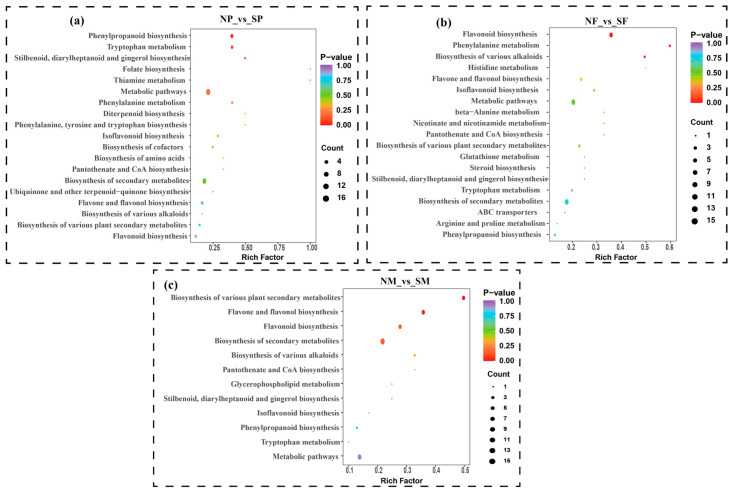
KEGG pathway enrichment bubble plots of differentially accumulated metabolites in (**a**) NP_vs_SP, (**b**) NF_vs_NF, and (**c**) NM_vs_SM (*p* < 0.05). Peel: (NP and SP), pulp: (NF and SF), and segment membrane: (NM and SM).

KEGG enrichment analysis identified pathways associated with differentially accumulated metabolites across tissue comparison groups (NP_vs_SP, NF_vs_SF, and NM_vs_SM) (*p* < 0.05; Figure 5).

These comparisons reflect metabolite regulation in seeded tissues (SP, SF, and SM) relative to their seedless counterparts. In NP_vs_SP, enriched pathways included phenylpropanoid biosynthesis (ko00940), tryptophan metabolism (ko00380), and stilbenoid, diarylheptanoid and gingerol biosynthesis (ko00945; *p* < 0.05), with additional enrichment in flavonoid biosynthesis (ko00941) (Figure 5a). The relatively high rich factor for phenylpropanoid-related pathways suggests pronounced phenolic metabolite differences in seeded peel (SP) compared with seedless peel (NP). In NF_vs_SF, flavonoid biosynthesis and phenylalanine metabolism (ko00360) showed strong enrichment (*p* < 0.05), together with the biosynthesis of various alkaloids (ko00996; *p* < 0.05) and isoflavonoid biosynthesis (ko00943) (Figure 5b), suggesting coordinated difference in flavonoid- and amino acid-derived secondary metabolites in seeded pulp (SF). In NM_vs_SM, enrichment was primarily observed in the biosynthesis of various plant secondary metabolites (ko00999), flavone and flavonol biosynthesis (ko00944), and flavonoid biosynthesis (*p* < 0.05), with additional contribution from phenylpropanoid biosynthesis (Figure 5c). This pattern indicates significant phenotype-associated metabolic reprogramming in seeded membrane tissue (SM). Across tissues, secondary metabolite-related pathways, particularly phenylpropanoid and flavonoid biosynthesis, were consistently enriched, supporting phenotype-dependent remodeling of phenolic and flavonoid metabolic networks in mature Ougan fruit.

**Figure 6:**
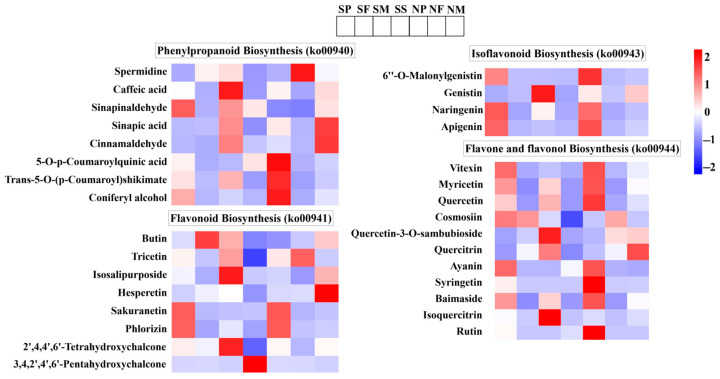
Graphical illustration of DAMs annotated to enriched secondary metabolite biosynthesis-related pathways across peel (SP and NP), pulp (SF and NF), segment membrane (SM and NM), and seeds (SS) of seeded (S) and seedless (N) Ougan fruits based on their expression values. Red indicates relatively higher abundance, and blue indicates relatively lower abundance of DAMs.

Based on the KEGG enrichment results, phenylpropanoid- and flavonoid-related pathways were further examined to evaluate metabolite accumulation patterns across tissues (Figure 6).

**Figure 7:**
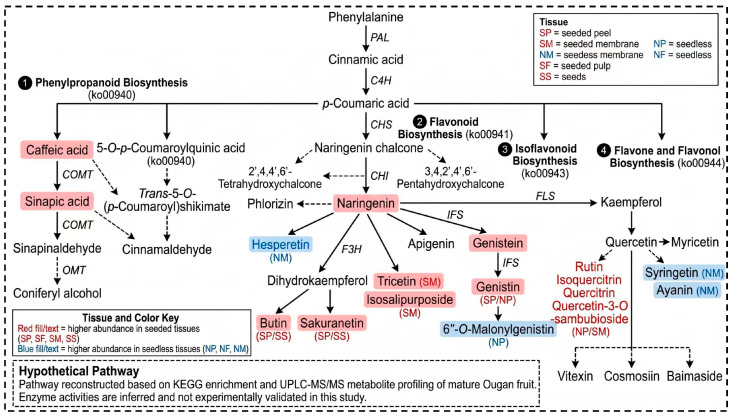
Hypothetical biosynthetic pathway of phenylpropanoid and flavonoid metabolism reconstructed from KEGG enrichment analysis and UPLC-MS/MS metabolite profiling of seeded and seedless *Citrus suavissima* tissues at commercial harvest maturity. Red shading indicates relatively higher abundance in seeded tissues (SP, SF, SM, and SS); blue shading indicates relatively higher abundance in seedless tissues (NP, NF, and NM). Solid arrows indicate established enzymatic steps; dashed arrows indicate inferred or hypothetical conversions. Enzyme activities are inferred from metabolite profiles and have not been experimentally validated in this study. Enzyme abbreviations: PAL, phenylalanine ammonia-lyase; C4H, cinnamate 4-hydroxylase; CHS, chalcone synthase; CHI, chalcone isomerase; F3H, flavanone 3-hydroxylase; FLS, flavonol synthase; IFS, isoflavone synthase; FNS, flavone synthase; COMT, caffeic acid O-methyltransferase; OMT, O-methyltransferase.

Within phenylpropanoid biosynthesis (ko00940), sinapinaldehyde and coniferyl alcohol accumulated strongly in peel tissues (SP and NP), whereas caffeic acid was markedly enriched in seeded membrane tissue (SM). In contrast, sinapic acid and cinnamaldehyde displayed pronounced accumulation in seedless membrane tissue (NM), suggesting differential distribution of downstream phenylpropanoid intermediates. In flavonoid biosynthesis (ko00941), butin and sakuranetin were highly accumulated in peel (SP) and seeds (SS) of seeded fruit, while tricetin and isosalipurposide showed peak levels in seeded membrane tissue (SM). Hesperetin was strongly enriched in seedless membrane tissue (NM), suggesting phenotype-associated difference in flavanone derivatives. In isoflavonoid biosynthesis (ko00943), genistin, naringenin, and apigenin accumulated predominantly in peel tissues (SP and NP), whereas 6-O-malonylgenistin showed higher abundance in seedless peel (NP). In flavone and flavonol biosynthesis (ko00944), quercetin, myricetin, syringetin, and rutin were enriched in seedless peel (NP), while quercetin-3-O-sambubioside and isoquercitrin accumulated prominently in seeded membrane tissue (SM). Overall, phenylpropanoid and flavonoid metabolites exhibited pronounced tissue specificity, with peel (SP and NP) and membrane (SM and NM) showing the highest metabolic activity. These results reveal tissue-specific specialized metabolite patterns associated with the seeded and seedless fruit phenotype. To contextualize the tissue-specific and phenotype-associated metabolite accumulation patterns within their biosynthetic framework, a hypothetical phenylpropanoid and flavonoid biosynthesis pathway was reconstructed based on the KEGG enrichment results and is presented in Figure 7. The pathway integrates the major differentially accumulated metabolites identified across peel, pulp, and segment membrane tissues within the four enriched KEGG pathways (ko00940, ko00941, ko00943, and ko00944), with metabolites color-coded according to their relative abundance in seeded (red) versus seedless (blue) tissues.

## 4. Discussion

In the present study, a comprehensive UPLC-MS/MS-based metabolomic approach was employed to characterize tissue-resolved metabolic variation between seeded and seedless cultivar forms of Ougan fruits sampled at commercial harvest maturity. Comparative pathways enrichment analysis showed that phenylpropanoid biosynthesis, phenylalanine metabolism, and flavonoid biosynthesis differed across peel (SP and NP), pulp (SF and NF), and segment membrane (SM and NM) tissues. Major discriminating metabolites included flavonoids, phenylpropanoids, and alkaloid derivatives, representing dominant classes of citrus secondary metabolism in Ougan. These findings demonstrate strong tissue specificity and phenotype-associated differences in phenolic and flavonoid metabolic profiles at fruit maturity.

Metabolic classification identified flavonoids as the predominant group, indicating substantial investment in phenylpropanoid-derived specialized metabolism, a hallmark of citrus fruit chemistry associated with antioxidant capacity, pigmentation, and flavor traits [3,4]. Phenolic acids, followed by alkaloids and lignans/coumarins, further highlight aromatic defense and quality-associated metabolites. Phenolic acids contribute to redox buffering and sensory attributes, whereas citrus alkaloids, including synephrine-type protoalkaloids, and coumarins are recognized for protective and bioactive functions [19].

In contrast, the relatively low proportions of steroids and tannins indicate that these classes are minor components of the mature Ougan metabolome, consistent with citrus profiles dominated by flavonoids and phenolics [3,4]. Overall, the dominance of flavonoids and phenolic acids underscores their central contribution to fruit quality and antioxidant potential [4,7].

The pronounced tissues specialization observed among the 1333 annotated metabolites aligns with the preferential allocation of defense- and quality-related metabolites to outer fruit tissues. Peel enrichment in protoalkaloids and secondary metabolites is expected because citrus peel functions as a primary protective barrier and bioactive reservoir. In seeded peel (SP), the dominance of synephrine supports this interpretation, as p-synephrine is a characteristic citrus protoalkaloids enriched in peel tissues [20,21]. High abundance of 5,7,5′-trimethoxy-3,4-methylenedioxyflavonoid is consistent with peel-biased accumulation of polymethoxyflavones (PMFs), which are associated with citrus antioxidant and bioactive properties [14]. The prominence of petrolactam in both SP and NP suggests a stable contribution of lactam-type metabolites, although its biological origin in citrus requires further validation [22]. The presence of aracarpene 1, a pterocarpene reported as a defense-associated metabolite in other plant systems, may reflect protective chemistry in peel tissues [23,24]. The tissue-specific accumulation patterns observed across Ougan fruit reflect the distinct biological roles of each tissue. Peel enrichment in synephrine, polymethoxyflavones, and phenylpropanoid derivatives is consistent with the role of this tissue as the primary chemical and physical barrier against biotic stresses, UV radiation, and pathogen attack, with these compounds functioning as deterrents and antioxidants [20,21]. Segment membrane enrichment in narirutin, isoquercitrin, and flavonoid glycosides reflects the structural and antioxidant protective function of this tissue toward the juice vesicles, consistent with the established roles of flavonoid glycosides in redox buffering and cell wall stabilization [24,25]. Seed-specific accumulation of limonin is consistent with its well-established role as a chemical deterrent against seed predation and herbivory [9,10]. These tissue–function relationships suggest that the metabolic architecture of Ougan fruit is shaped not only by biosynthetic capacity but also by tissue-specific functional demands.

In pulp tissues, enrichment of amide- and alkaloid-type metabolites is consistent with citrus pulp serving as a reservoir of soluble bioactive compounds contributing to taste and nutritional quality. The high abundance of N-(3-hydroxy-4-methoxyphenethyl)-4-hydroxybutanamide in seeded pulp (SF) aligns with phenolamide/arylalkylamide metabolites associated with stress-responsive and antioxidant-related metabolism [13,26]. The recurrent detection of pterolactam in both SF and NF further suggests a consistent presence of heterocyclic metabolites [15,27]. Conversely, 4-nitrophenol is widely documented as an environmental pollutant; therefore, its detection may reflect exogenous exposure, annotation ambiguity, or matrix effects and requires targeted confirmation [25,28]. Enrichment of hesperetin-7-O-glucoside in seedless pulp (NF) is consistent with citrus flavonone dominance and antioxidant properties [29,30].

Segment membrane tissues accumulated abundant flavonoid glycosides, consistent with their structural and protective roles. In seeded membrane (SM), elevated synephrine and narirutin reflect alkaloid and flavanone enrichment; narirutin is widely reported as a major citrus flavanone glycoside [1,31]. As in pulp, the high signal of 4-nitrophenol warrants cautious interpretation [32,33]. Greater accumulation of narirutin and isoquercitrin in seedless membrane (NM) indicates phenotype-associated variation in flavonoid glycosylation [34]. Seeds (SS) were characterized by high limonin, consistent with established enrichment of limonoids in citrus seeds and their contribution to bitterness [9,10]. Co-accumulation of luteolin-7-O-(6-malonyl) glucoside further supports seed-associated enrichment of complex flavonoid glycosides [1].

Differential metabolite analysis (Log_2_FC) indicates that the seeded–seedless fruit phenotype is associated with targeted differences in phenylpropanoid and flavonoid metabolism. The strong relative increase of 6-hydroxykaempferol-3,6-O-diglucoside in seeded peel (SP) is consistent with enhanced accumulation of flavonol glycosides, recognized as antioxidant and photoprotective end products of the phenylpropanoid pathway [14,35]. Increased 4-hydroxybenzoic acid further supports intensified phenolic metabolism, as hydroxybenzoates derive from phenylpropanoid turnover and contribute to antimicrobial and defense-related functions [36,37]. Elevated tamarixetin, a methylated quercetin derivative, aligns with enhanced antioxidant flavonoid chemistry [38]. Conversely, reduced delphinidin-3-O-rutinoside-7-O-glucoside suggests lower allocation toward delphinidin-based anthocyanins, pigments associated with coloration in the late branches of flavonoids [39,40]. In NF_vs_SF, increased N-sinapoylhydroxycoumarin in seeded pulp (SF) is consistent with greater accumulation of hydroxycinnamic acid amides (HCAAs), a phenylpropanoid-derived class linked to defense and oxidative stress buffering [41,42]. Increased narcissin (isorhamnetin-3-O-rutinoside) suggests enhanced flavonol glycosylation, a mechanism that stabilizes and stores antioxidant flavonoids in edible tissues [43]. Increased petunidin-3-O-(6-O-p-coumaroyl)-glucoside suggests stimulation of anthocyanin-related metabolism, as acylation and glycosylation improve pigment stability and antioxidant function [44,45]. In contrast, decreased nagilactone C glucoside reflects lower representation of terpenoid lactone-type metabolites [8,46].

In NM_vs_SM, increased isosaponarin in seeded membrane (SM) suggests enhanced accumulation of flavonoid glycosides, consistent with membrane tissues functioning as sites of flavonoid storage and antioxidant activity [47,48]. Increased 7-hydroxy-costol-malonyl glucoside further indicates elevated glycosylation/acylation of specialized metabolites, a common stabilization strategy [49]. Conversely, reduced hyperin (hyperoside; quercetin-3-O-galactoside) suggests altered flavonol allocation within membrane tissues [50]. Consistent enrichment of phenylpropanoid and flavonoid biosynthesis across NP_vs_SP, NF_vs_SF, and NM_vs_SM supports phenotype-associated shifts in phenolic flux, while phenylalanine and tryptophan metabolism (ko00380) suggest altered aromatic precursor availability [2,14,41]. Accumulation of sinapinaldehyde and coniferyl alcohol in peel (SP/NP) indicates enhanced hydroxycinnamyl-aldehyde/alcohol branch activity associated with monolignol-related protective metabolism [51,52]. Enrichment of caffeic acid in seeded membrane (SM) supports enhanced antioxidant phenolic pools [53,54], whereas higher sinapic acid and cinnamaldehyde levels in seedless membrane (NM) reflect phenotype-dependent distribution of downstream phenylpropanoid intermediates [55,56]. Within flavonoid biosynthesis, elevated butin and sakuranetin in seeded peel (SP) and seed (SS) indicate enhanced stress and defense-associated flavonoid deployment; notably, sakuranetin functions as a flavonoid phytoalexin [57,58]. Accumulation of tricetin and isosalipurposide in seeded membrane (SM) supports tissue-specific antioxidant glycoside storage [59]. Enrichment of hesperetin in seedless membrane (NM) reflects citrus flavonone dominance and phenotype-associated variation in flavonone pools [30,60].

In the isoflavonoid branch, accumulation of genistin, naringenin, and apigenin in peel (SP/NP) aligns with the role of citrus peel as a major flavonoid reservoir [11,61]. Increased 6-O-malonyulgenistin in seedless peel (NP) highlights enhanced malonylation, which improves flavonoid stability and vacuolar sequestration [60]. Enrichment of quercetin, myricetin, syringetin, and rutin in NP indicates phenotype-associated shifts toward flavonol-type antioxidants [1,61], whereas accumulation of quercetin-3-O-sambubioside and isoquercitrin in seeded membrane (SM) suggests tissue-specific glycosylation and stabilization of quercetin derivatives [62,63]. Collectively, these results demonstrate tissue-dependent differences in flavonoid glycosylation and phenylpropanoid-derived antioxidant pools across mature Ougan fruit tissues. Overall, tissue specialization defines the core metabolic architecture of Ougan fruit, while the seeded–seedless phenotype is associated with targeted differences in phenylpropanoid, flavonoid, and alkaloid pathways, resulting in differential distribution of antioxidant- and quality-associated secondary metabolites at maturity. The metabolic shifts observed between seeded and seedless tissues may be partly explained by the developmental consequences of seedlessness. The seedless Ougan cultivar is a naturally occurring bud-sport mutant characterized by male sterility and pollen abortion, consistent with other seedless citrus mutants [12,13]. The absence of seeds during fruit development likely alters phytohormone dynamics, particularly auxin and gibberellin gradients, which are known regulators of flavonoid and phenylpropanoid metabolism in developing citrus fruit. Seed-derived hormonal signals are well established as drivers of metabolite accumulation in surrounding tissues; their absence in seedless fruit may therefore directly account for the differential distribution of flavanones, flavonol glycosides, and malonylated isoflavonoids observed between seeded and seedless tissues. Elucidating the precise molecular mechanisms underlying these shifts will require integration of transcriptomic and hormonal profiling data, which we propose as a priority for future multi-omics studies.

## 5. Conclusions

This study was limited to a single developmental stage, orchard, and growing season (November 2024), and metabolite accumulation may vary under different climatic conditions. In addition, the study did not provide direct evidence of metabolic flux or pathway regulation, and some compounds (e.g., 4-nitrophenol), as well as key differential metabolites, require targeted validation. Further integration of UPLC-MS/MS data and pathway-based analyses will also help improve biological interpretation in future studies. Nevertheless, this work represents the first tissue-resolved metabolomic characterization of mature Ougan fruit and the first comparative metabolomic baseline between seeded and seedless C. suavissima phenotypes, providing a valuable foundation for future multi-season, multi-omics studies and marker-assisted breeding programs.

This study provides a comprehensive tissue-resolved metabolomic profiling of seeded and seedless Ougan fruits at commercial harvest maturity using UPLC-MS/MS. The results demonstrate that tissue specialization is the primary determinant of metabolic architecture, with peel and segment membrane tissues exhibiting a high relative abundance of flavonoids and phenylpropanoid-derived compounds. In addition, the seeded–seedless phenotype was associated with tissue-dependent differences in phenylpropanoid, flavonoid, alkaloid, and limonoid pathways, leading to differential distribution of antioxidant- and quality-associated metabolites. Key discriminating compounds, including flavonol glycosides, hydroxycinnamic acid derivatives, synephrine, and limonin, highlight coordinated shifts in secondary metabolism linked to fruit phenotype. Collectively these findings enhance understanding of the biochemical basis of Ougan fruit quality and provide a metabolic foundation for future breeding.

## Data Availability

The original contributions presented in this study are included in the article/Supplementary Materials. Further inquiries can be directed to the corresponding author.

## Supplementary Materials

The following supporting information can be downloaded at: https://www.mdpi.com/article/10.3390/cimb48060596/s1.
